# Lessons from a comparison of immuno-chromatographic and chemiluminescent micro-particle immunoassay in the diagnosis of syphilis

**DOI:** 10.1186/s13104-018-3808-5

**Published:** 2018-10-04

**Authors:** Sepiso K. Masenga, Mercy Mweemba, Annie Kachele, Yahns Chalubemba, Paul Toloka, Benson M. Hamooya

**Affiliations:** 1 Pathology Laboratory Department, Research Section, Livingstone Central Hospital, Akapelwa Street, P.O. Box 60091, Livingstone, Zambia; 2 Department of Biomedical Sciences, School of Health Sciences, University of Zambia, P.O. Box 50110, Lusaka, Zambia; 3 School of Public Health, University of Zambia, P.O. Box 50110, Lusaka, Zambia; 4 Chikankata College of Biomedical Sciences, Private Bag Sector 2, Mazabuka, Zambia

**Keywords:** SD Bioline, Immunoassay, Architect syphilis, *Treponema pallidum*

## Abstract

**Objective:**

To synthesize lessons from comparison of results obtained from the immuno-chromatographic SD Bioline testing method and the chemiluminescent micro-particle immunoassay Architect in the diagnosis of syphilis at Livingstone Central hospital laboratory.

**Results:**

The specificity and sensitivity of SD Bioline syphilis 3.0 against the chemiluminescent immunoassay using the Architect syphilis *Treponema pallidum* (TP) was 85.3% and 91.3% respectively with substantial agreement between the two test methods (88%, ĸ  = 0.76; p < 0.0005). We recommend further comprehensive study with a larger sample size and clinical details to ascertain the validity of our findings. We also recommend using a non-treponemal test with the current treponemal tests being used to aid diagnosis.

## Introduction

The laboratory serologic diagnosis of syphilis can be complicated [1] especially with limited diagnostic tools owing to the natural course of the infection which is characterized by periods with and without clinical manifestations [1]. Furthermore, the disease stages show variable sensitivity and specificity for some test methods in detecting *Treponemal pallidum* (TP) antibodies [1, 2]. It is therefore mandatory to interpret laboratory results on the basis of patient history and physical examination findings [3]. The traditional screening of syphilis recommended by the Center for Disease Control and Prevention (CDC) is the use of a nontreponemal test, such as rapid plasma reagin (RPR) [4]. However, the use of chromatographic and chemiluminescence immunoassay (CIA) treponemal test methods for screening syphilis is empirical and gaining acceptability [5]. It is important and mandatory to evaluate the diagnostic performance of laboratory test methods in every laboratory so as to instill confidence in the clinician and patient regarding the reliability of laboratory results [1]. Several reports have imaged that have compared the SD Bioline syphilis test with other treponemal methods, stressing the importance of conducting pre-clinical and -laboratory performance and applicability of all testing methods for syphilis diagnosis [6, 7]. At Livingstone Central hospital, which is based in the Southern Province of Zambia, we use the SD Bioline syphilis test only (recently introduced), as an index test to aid in the diagnosis of syphilis for patients while Architect syphilis TP is specifically used for screening blood donors. The disease burden of syphilis in our region is unknown owing to paucity of studies. We decided to perform an exploratory study whose objective was to compare the SD Bioline syphilis test performance against the Architect syphilis TP. This was prodded by the clinicians’ concern for the high TP positivity rate recorded for the months, March to May, 2017 using the SD Bioline syphilis test.

## Main text

### Methods

#### Study design

This was a cross-sectional study conducted at the Livingstone Central hospital laboratory research section.

#### Setting and sample size

Data collection was planned after the index test but before the reference standard was performed. We collected data for the SD Bioline syphilis 3.0 positivity rate for the months of March, April and May, from the laboratory information system as shown in Table 1.

**Table 1:** Positivity rate of suspected syphilis samples using SD Bioline test

SD Bioline test	March	April	May	Total
Positive	44 (19.8%)	46 (21.5%)	26 (11.4%)	116 (14.9%)
Negative	222 (80.2%)	214 (78.5%)	229 (88.6%)	665 (85.1%)
Total	266 (100%)	260 (100%)	255 (100%)	781 (100%)

We then randomly selected 100 samples from the syphilis suspected specimens that tested reactive and non-reactive (1:1) on SD Bioline which were compared with the reference standard, Architect syphilis TP. We used the available electronic laboratory information system to confirm the clinical suspicion for syphilis. Samples had been collected through venipuncture into plain glass vacutainers containing no clot activators, anticoagulants, preservatives or separator materials which and were stored at 2–8 °C for a minimum of 45 min before centrifugation to extract serum for testing.

#### Test methods

The SD Bioline syphilis 3.0 (Standard diagnostics, INC. Korea) is a solid phase immunochromatographic assay for the qualitative detection of antibodies of all isotypes [Immunoglobulin G (IgG), Immunoglobulin M (IgM), Immunoglobulin A (IgA)] against TP. It is pre-coated with recombinant TP antigens that bind to the TP antibodies, if present in the patient sample to produce a visible line. In this study, SD Bioline syphilis 3.0 is the index test.

The Architect syphilis TP (Abbott GmbH & Co. KG, Germany) is a two-step chemiluminescent microparticle immunoassay (CIA or CMIA) for the qualitative detection of IgM and IgG antibodies to TP intended to aid in the diagnosis of syphilis infection and as a screening test in blood donors to prevent transmission of TP to recipients. The method and principle of analysis are described in detail elsewhere [8]. Architect syphilis TP was used as our reference standard.

#### Bias

Since the sample size was not calculated based on any hypotheses but selected based on the availability of reagents, we most likely introduced selection bias by selecting 50 positives and 50 negative samples. However, the selection of these samples was random to minimize selection bias.

#### Statistical methods

Descriptive statistics was used to summarize data while Chi square or fishers exact test compared results for the two diagnostic testing methods. A concordance test assessed the degree of agreement between the two tests and receiver operating curve (ROC) to evaluate the benefit of using the index test.

#### Reporting guidelines

We used the Standards for Reporting of Diagnostic Accuracy Studies (STARD) [9].

### Results

Total number of syphilis suspected samples collected and tested between March and May were 781 (Table 1) of which 116 (14.9%) tested positive with SD Bioline syphilis rapid kit. There was a drop, in positivity rate for the month of May compared to the first 2 months.

50 samples that tested negative and positive respectively, on SD Bioline syphilis rapid kit were retested on Architect and the diagnostic tests then evaluated (Table 2).

**Table 2:** False positivity and false negative rates of SD Bioline compared with architect syphilis test

		Architect TP		*p*-value
		Positive	Negative	
SD Bioline	Positive	42 (91.3%)	8 (14.8)	< 0.0005
	Negative	4 (8.7%)	46 (85.2%)	
Total		46 (100%)	54 (100%)	

p-value, Fishers’ exact test used

As shown in Table 2, the false positive rate for SD Bioline was 14.8% with a sensitivity of 91.3% while the false negative rate was at 8.7% with a specificity of 85.2%. There was a significant difference between the two tests p < 0.0005. Positive and negative likelihood ratios of 6.2 and 0.1 were reported respectively (data not shown).

The positive and negative predictive values were 84% and 93% at prevalence of 54% (expected). However, using actual point prevalence of 15.3%, the positive and negative predictive values were 53.7% and 46.9%.

### Roc curve for SD Bioline against the Architect test

We performed a receiver operator curve (ROC) analysis (graph not shown) yielding an area under the curve of 0.88, (standard error, 0.032; 95% confidence interval [0.81, 0.95] p < 0.001).

We also performed a concordance test (Table 3 below) to assess the degree of agreement between the two tests.

**Table 3:** Cohen’s kappa (ĸ) results showing a good degree of concordance between SD Bioline and Architect tests

Agreement	ĸ	Standard error	p value
88%	0.760	0.0997	p < 0.0005

## Discussion

The specificity (85.3%) and sensitivity (91.3%) of SD Bioline against the Architect in our study were significantly low especially that SD Bioline and the Architect sensitivity and specificity compared to several non-treponemal tests can go as low as 95% and 98% respectively [10]. However, in another study, the sensitivity of SD Bioline was comparable (92%) [11], moreover a study conducted in Tanzania found the test to be 79% sensitive and 96% specific [12].

The marginal distributions of test ratings did not indicate prevalence or bias problems, suggesting that Cohen’s kappa was an appropriate index of inter-rater reliability, so we performed an inter-rater reliability test using Cohen’s kappa (ĸ) to determine if there was agreement between the two diagnostic tests in our study. The results (88%, ĸ = 0.76; p < 0.0005) obtained indicate substantial agreement [13]. An almost perfect agreement (ĸ = 0.81–1.00) is the best every lab would want to achieve. However, the difference could be explained by the technicality involved in the performance of the two tests, the architect being fully automated, hence not prone to a lot of errors and expert interpretation as compared to the immunochromatographic method used in the SD Bioline syphilis 3.0 which is prone to pre- and -analytical errors. Nonetheless, the scope of our study could not allow us to determine the real difference.

The clinical implications of our results in the diagnosis of syphilis can only be assessed with additional clinical information. It remains a standard that when interpreting laboratory results for syphilis, we must consider the clinical stage aided by patient history and physical examination. Without these, erroneous diagnosis and bad management decisions is the consequence [4, 14].

The laboratory implication from the results we obtained warrants a follow up study to investigate and determine the efficiency and application of the chemiluminescence immunoassay and immunochromatographic assay in syphilis diagnosis as it is difficult to prove that positive or negative results obtained using these platforms are necessarily false [1, 15] hence the need for patient medical history including previous treatment for syphilis, and history of sexual risk factors [1, 2]. Additionally, we recommend adding a non-treponemal test like RPR and possibly implement reverse algorithm in syphilis diagnosis. The use of both non-treponemal and treponemal tests for diagnosis is warrantable for laboratories [1]. It also means that the laboratory needs to obtain complete clinical information for accurate interpretation of results to guide the clinician in managing the patients appropriately [16]. The test methods involved in our study are treponemal tests. It is well known that these tests can remain positive almost for life in previously infected individuals regardless of treatment [1] while antibodies measured in non-treponemal tests do disappear after some years [17]. It is therefore, vital, to use both treponemal and non-treponemal tests, to differentiate previous infection from current [17]. However, we also know that a confirmed positive serological test result does not differentiate disease stage and we may not tell if the infection is past or current in some cases [17]. Given the limitations of TP tests, we can still use them to make accurate diagnosis aided by clear patient history and evidence from physical examination [17].

For primary syphilis, the recommended tests to perform include direct examination, non-treponemal tests, and treponemal tests and since there is no single optimal test, a combination of these is required to aid in the diagnosis [1, 2]. Detection of TP in lesions is definitive evidence of syphilis but a negative result does not rule out syphilis [18]. PCR-based tests have a high reliability [1, 18]. In the first 2–3 weeks, serology may not be positive in some cases, and in early primary syphilis, treponemal tests maybe recommended [19]. When there is evidence of a genital ulcer at physical examination and a non-treponemal test is reactive, this may not necessarily indicate primary syphilis [17] and in such cases a repeat test over a two to 12 week period may be required to rule out syphilis [17]. In secondary syphilis, the use of direct physical examinations, and use of both non-treponemal tests and treponemal tests are still recommended [17, 19]. In persons with a history of syphilis, a fourfold increase in titer when using quantitative methods provides presumptive diagnosis of secondary syphilis [17]. In latent syphilis, as in all stages, it is recommended to use both treponemal and non-treponemal tests [17]. However, non-treponemal tests are reactive in early latent syphilis though the sensitivity declines over time [1, 17]. Prevalence information of syphilis in a population is cardinal for interpretation, as in low prevalence populations, false-positive results may be common with both treponemal and non-treponemal tests [17].

As a general rule, a reactive treponemal test in the absence of a reactive non-treponemal test may require confirmation [17]. For tertiary syphilis, non-treponemal and treponemal tests are recommended [15, 17, 19]. In this stage approximately 30% may not be reactive using non-treponemal tests, while treponemal tests are reactive almost always [17]. Therefore, we think treponemal tests should always be considered [17].

## Conclusion

The specificity and sensitivity of the immunochromatographic assay, SD Bioline syphilis 3.0 against the chemiluminescent immunoassay, Architect syphilis TP was 85.3% and 91.3% respectively with substantial agreement between the two test methods. However, we recommend further comprehensive study with a larger sample size and clinical details to ascertain the validity of our findings. We recommend using both treponemal and non-treponemal tests to aid diagnosis of syphilis.

## Limitations

Because we did not have access to clinical information, our study had limited variables needed for a comprehensive comparison. For example, we needed to compare our results to clinical findings and as well, compare with a non-treponemal test in order to provide an accurate conclusion on the implications of the false positive and false negative rates.

Our cohort was not well characterized to draw useful conclusions with clinical and laboratory implications. However, we want to use the lessons from our findings to propose for a well characterized follow-up study.

False-positive reactions can occur with treponemal tests but this is less common than with nontreponemal tests [17]. Examples of conditions were false positive tests are reported include but not limited to advancing age, brucellosis, cirrhosis, hyperglobulinemia, malaria, pregnancy, systemic lupus erythematosus et cetera [17]. Our study lacked information on these factors that have implications on the interpretation of TP laboratory results.

## Abbreviations

CDC: Centres for Disease Control and Prevention
CIA OR CMIA: chemiluminescence immunoassay
IgA: Immunoglobulin A
IgG: Immunoglobulin G
IgM: Immunoglobulin M
ROC: receiver operating curve
RPR: rapid plasma reagin
STARD: Standards for Reporting of Diagnostic Accuracy Studies
TP: *Treponemal pallidum*
